# Interventions for perinatal borderline personality disorder and complex trauma: a systematic review

**DOI:** 10.1007/s00737-023-01313-4

**Published:** 2023-04-20

**Authors:** Alexandra May, Ryan Balzan, Anne Sved Williams, Tracey D Wade, Sarah Marie Paranjothy

**Affiliations:** 1 Flinders Institute for Mental Health and Wellbeing and Blackbird Initiative, College of Education, Psychology, and Social Work, Flinders University, Adelaide, South Australia Australia; 2 University of Adelaide, Adelaide, Australia; 3 Women’s and Children’s Health Network, North Adelaide, South Australia Australia

**Keywords:** Borderline personality disorder, Complex post-traumatic stress disorder, Complex trauma, Perinatal, Intervention

## Abstract

Perinatal borderline personality disorder (BPD) and complex post-traumatic stress disorder (cPTSD) are associated with significant impairment to interpersonal functioning, and risk of intergenerational transmission of psychopathology. Evaluation of interventions, however, is scarce. To date, no systematic review has addressed interventions for perinatal BPD, cPTSD, and associated symptomatology. Given the modest evidence to support informed clinical guidelines, the objective of this systematic review is to synthesise the literature on interventions for perinatal BPD and cPTSD, and to generate future directions for research. A comprehensive literature search following PRISMA guidelines was conducted in PsycInfo, MEDLINE, Emcare, Scopus, and ProQuest Dissertations and Theses Global databases. Seven original studies were included, of which only two were randomised controlled trials, using less intensive comparison conditions. Results suggest an association between Dialectical Behavioural Therapy (DBT) group skills training, a multimodal therapeutic approach at a Mother-Baby Unit (MBU), and Child-Parent Psychotherapy with improved perinatal mental health outcomes and remission of symptoms. MBU admission and home-visiting programs were associated with healthy postpartum attachment relationships. Home-visiting programs and DBT group skills were additionally associated with improved maternal parenting capabilities. Conclusions to inform clinical guidelines are limited by a lack of credible comparison conditions, and low quantity and quality of evidence. The feasibility of implementing intensive interventions in real-world settings is dubious. Hence, it is suggested that future research considers utilising antenatal screening to identify at-risk mothers, and the implementation of early intervention, using robust designs that can inform robust conclusions.

## Introduction

The perinatal period, widely regarded as the time of pregnancy to 12 months postpartum, involves immense change, novel challenges, and emotional lability (Chlebowski, 2013). Previous coping strategies may prove inadequate or inappropriate when faced with sleep deprivation, a crying infant, physical pain, role transformation, and relationship strain (Geerling et al., 2019; Sved Williams & Apter, 2017; Wilson & Donachie, 2018). A serious psychiatric disorder, borderline personality disorder (BPD) is characterised by intense emotions, unstable interpersonal relationships, and behavioural impulsivity (American Psychiatric Association, 2013). BPD symptomatology may exacerbate postpartum challenges in new mothers (Sved Williams et al., 2018; Yelland et al., 2015; Dunn et al., 2020).

Highly comorbid with BPD and with comparable disturbances in emotion regulation, interpersonal relations, and self-concept (Ford & Courtois, 2021), is complex post-traumatic stress disorder (cPTSD) (van Dijke et al., 2018). Some distinctions are however noted. BPD is categorised as a personality disorder (American Psychiatric Association, 2013; World Health Organization, 2019) and involves extreme sensitivity to perceived abandonment. In contrast, cPTSD is considered a disorder related to stress (World Health Organization, 2019) and involves hypervigilance for being harmed (van Dijke et al., 2018). Occasionally used interchangeably with cPTSD within the literature is “complex trauma”; however, it is noted that although complex trauma is defined by repeated and prolonged traumatic events, it is not pathological. Nevertheless, although cPTSD/complex trauma and BPD are separate constructs with distinct diagnostic categories, research supports extensive symptom overlap (Jowett et al., 2020). In consideration of this and comparable etiology, “BPD/trauma” will henceforth be used in the present review to encompass BPD and cPTSD/complex trauma.

Mothers with BPD are more likely to experience compromised obstetric outcomes including emergency caesarean delivery and preterm birth (Blankley et al., 2015; di Giacomo et al., 2020; Pare-Miron et al., 2016). Post-childbirth, maternal BPD can interfere with parenting efficacy through decreased emotion recognition, inconsistency, insensitivity, overprotection, hostility, and abuse (Chlebowski, 2013; Crandell et al., 2003; Newman & Stevenson, 2005; Sved Williams et al., 2018; White et al., 2011). Volatile maternal behaviours can increase the risk of the infant developing a disorganised attachment style, characterised by contradictory attachment behaviours (Hobson et al., 2005). Initially producing profound consequences for infant social-emotional development (Chlebowski, 2013; Hobson et al., 2005), subsequent problematic interpersonal relations can perpetuate the cycle of intergenerational transmission of psychopathology (Wilson & Donachie, 2018). In comparison to children of mothers with no psychopathology, depression, or other personality disorders, children of mothers with BPD are more likely to develop BPD (Chlebowski, 2013; Eyden et al., 2016); depression, anxiety, low self-esteem (Barnow et al., 2006), and a dysregulated sense of self and emotions (Macfie & Swan, 2009).

Presentation of perinatal BPD ranges from 2.0 to 35.2%, and BPD symptomatology ranges from 9.7 to 34% (Prasad et al., 2022). Yet, screening and intervention are not offered in routine antenatal care. Furthermore, despite the risks to mothers and infants, evaluation of interventions addressing perinatal BPD/trauma has been overlooked in the research literature. Although research on postpartum depression and anxiety continues to expand, consideration of comorbid BPD/trauma could further substantiate findings on these prevalent perinatal psychiatric disorders. At the time of conducting this review, we were unable to locate a systematic review or meta-analysis examining interventions for perinatal BPD/trauma. Hence, given the modest evidence to support informed clinical guidelines, the objective of this systematic review is to synthesise the literature on interventions for perinatal BPD/trauma, and to generate future directions for research.

## Methods

### Search strategy

The present review was conducted and reported according to the evidence-based guidelines for reporting systematic reviews and meta-analyses (Moher et al., 2009). A literature search was conducted in databases PsycInfo, MEDLINE, Emcare, and Scopus for all years through to 22nd July 2022. The ProQuest Dissertations and Theses Global database was additionally searched for unpublished articles using the search terms in the title only. No restrictions were placed on publication type, date, or language. The review was registered with PROSPERO on 26th May 2021 (Ref: CRD42021257268). An expert librarian was consulted with regard to the search terms, with the following used in title and abstract: (“borderline personality disorder” OR “borderline states” or “complex PTSD” OR “borderline” adj4 (“disorder*” OR “trait*” OR “feature*” OR “pathology*” OR “etiology” OR “presentat*” OR “personality” OR “state*”) OR “BPD” or “emotional dysregulation” or “complex trauma” OR “complex PTSD”) AND (“perinatal period” OR “antepartum period” OR “postnatal period” OR “pregnancy” OR “infant*” OR “bab*” OR “neonat*” OR “newborn” OR “perinatal” OR “peripartum” OR “pregnan*” OR “antenatal” OR “antepartum” OR “prenatal” OR “postpartum” OR “postnatal”). The secondary search strategy involved hand searching any relevant articles identified within the primary search.

### Inclusion and exclusion criteria

To ensure inclusion of all relevant literature, the search included BPD, cPTSD, complex trauma, and emotional dysregulation. Other comorbid psychiatric disorders (i.e. attention deficit hyperactivity disorder and bipolar II disorder) were not included due to being beyond the scope of the current review. To further ensure inclusion of all relevant literature, specifications were not placed on intervention type. Exclusion criteria included case reports and reviews.

### Selection process and data collection

**Fig. 1:**
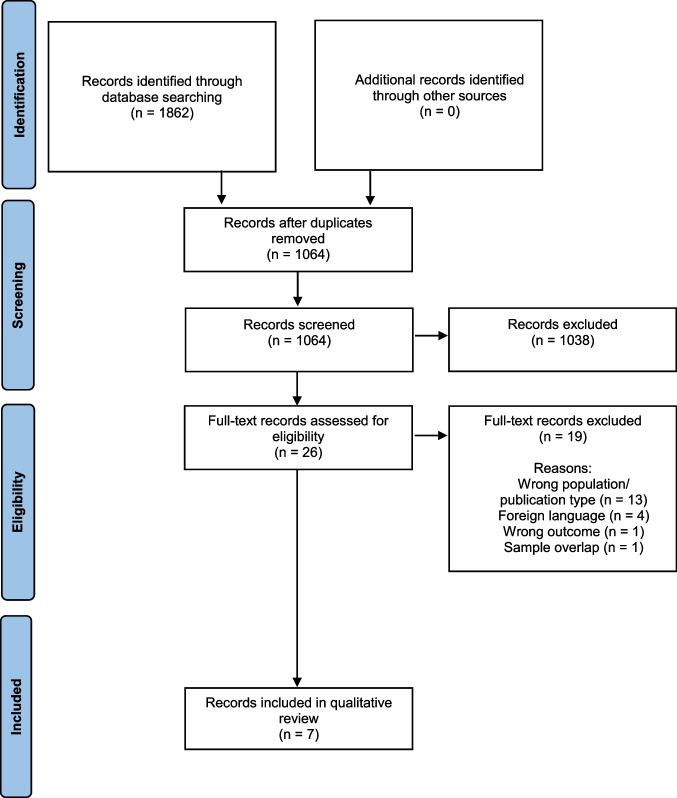
PRISMA flow chart of the selection process, specifying the number of records at the identification, screening, eligibility, and inclusion stages (Moher et al., 2009)

Search outputs from the four databases were cross referenced and duplicates were removed. Title and abstracts were assessed according to the inclusion criteria to determine relevance to the research question. Full texts of all remaining publications were then reviewed for eligibility for inclusion in the systematic review. Authors (AM and SP) consulted on each publication, with an agreement rate of 100% at full text. Figure 1 presents a flow diagram of the selection process.

### Risk of bias assessment

The Cochrane Effective Practice and Organisation of Care (EPOC) risk of bias (RoB) tool (2017) was used to assess the internal validity of each study according to seven domains. Each domain was classified as either low RoB (plausible bias unlikely to seriously alter the result), high RoB (plausible bias that seriously weakens confidence in the results), or unclear RoB (plausible bias that raises some doubt about the results).

## Results

### Selection of studies

The initial search yielded 1862 records, with 1064 records remaining after removing duplicates. Screening of each title and abstract then resulted in 1038 exclusions because they did not meet the inclusion criteria. Full texts of the remaining 26 records were assessed. Nineteen records were then excluded due to irrelevancy, including wrong population, publication type, and outcome, or restricted availability in a foreign language or sample overlap. One author confirmed an overlapping participant sample between two studies; hence, the record with the larger sample was retained (Sved Williams et al., 2021) with the smaller one (Sved Williams et al., 2018) being discarded. The present review included seven publications fulfilling the inclusion criteria.

### Study and participant characteristics

Seven journal articles were included in the review (Table 1), conducted in the past 17 years in western societies: Australia (*n* = 2), Canada (*n* = 1), United Kingdom (UK; *n* = 2), and USA (*n* = 2). Five studies were repeated measures designs, and two were randomised controlled trials (RCTs). Sample sizes ranged from *n* = 5 to *n* = 89, encompassing a total of 351 participants. Three interventions were delivered in postpartum samples, two interventions commenced with a pregnant sample and continued postpartum, and two interventions involved mixed samples consisting of pregnant and postpartum participants. Two RCTs involved adolescent samples.

**Table 1:** Characteristics of studies in the current review

**Author & year**	**Country**	**Design**	**Recruitment method**	**Sample size & population**	**Exclusion criteria**	**Pathology assessed**	**Assessment method**	**Intervention used**	**Comparators**	**Results**
Freethy et al. (2020)	UK	RM	Referred internally	• *n* = 5 women (1 pregnant, 4 postpartum) • Mean age = 26.00 years	N/A	• Depression • Anxiety • Parental bonding • Emotion regulation	• EPDS*** • GAD-7*** • PBQ*** • TAS-20****	12-weekly, 2-h DBT skills group program. Facilitated by a perinatal clinical psychologist, assistant psychologist and supported by a psychology placement student.	N/A	Significant decrease in levels of depression and anxiety. No statistically significant change found in emotion regulation and parent infant bonding. Qualitative feedback described that all participants found the sessions useful.
Lavi et al. (2015)	USA	RM	Referred by social workers	• *n* = 64 women (third trimester of pregnancy (53%)) • Mean age = 27.48 years • Predominantly Latina (86%)	• Insufficient cognitive capacity • Psychosis • Current use of alcohol/drugs	• Child-rearing attitudes • Depression • Post-traumatic stress symptoms (PTSS) • Current and past interpersonal trauma • Maternal-fetal attachment	• AAPI-2* • CES-D* • DTS* • LSC (pre-test) • MFA (pre-test)	Perinatal adaptation of Child-Parent Psychotherapy, with an average of 27 treatment sessions until the infant was 6 months old. Conducted by perinatal trained clinicians.	N/A	Significant decrease in levels of depression and post-traumatic stress symptoms. The greatest improvement was seen in women with low maternal-fetal attachment. Women also showed more positive child-rearing attitudes at the post- treatment assessment compared to pre-treatment levels.
Moran et al. (2005)	CA	RCT	Recruited during postpartum stay at hospital	• *n* = 50 women (postpartum) • Mean age = 18.42 years • Predominantly Caucasian (81%)	N/A	• Complex trauma • PTSD • Unresolved/disoriented attachment	• AAI (pre-test) • SSP (post-test) • MBQS***	8 home intervention sessions for approximately 1 h, when the infant is between 7 and 12 months of age. Facilitated by clinically trained visitors.	50 adolescent mother-infant dyads received 1 home intervention session.	Significantly more mother-infant dyads who received the intervention had secure attachment relationships when the infant was 12 months old, compared with the control group. More mothers in the intervention group maintained sensitivity at 24 months in comparison to the control group.
Sadler et al. (2013)	USA	RCT	Recruited from prenatal groups	• *n* = 60 women (pregnant, primiparous) • Mean age = 19.60 years • Predominantly Latina (62%)	• Non-English speaking • Not between 14–25 years of age • Active heroin or cocaine use • DSM-IV psychotic disorder • Major/terminal chronic condition	• Relationship quality/interaction • General mental health • Depression • Child abuse/neglect • Child health outcomes • Parental bonding • Reflective functioning • Infant attachment	• AMBIANCE (during) • BSI** • CES-D** • Child protective referral***** • Health record review***** • PBI (pre-test) • Parent development interview (post-test) • Pregnancy interview (pre-test) • SSP (during)	"Minding the Baby". An intensive home-visiting program based on mentalisation. Weekly visits for approximately 1 h, from the third trimester until the child is age 1, followed by biweekly visits until the child is age 2. Delivered by a team including a master’s degree level nurse and social worker.	45 mother-infant dyads received routine care as usual.	Intervention group more likely to be on track with immunisation schedules at 12 months and had lower rates of rapid subsequent childbearing. No significant differences between likelihood of referral to child protective services. Mother-infant interactions less likely to be disrupted at 4 months for teenaged mothers in the intervention group, and all infants more likely to be securely attached at 1 year in the intervention group. Parental reflective functioning improved for high-risk mothers at 24 months in comparison to controls.
Sved Williams et al. (2021)	AUS	RM	Referred by clinician	• *n* = 69 mother-child dyads (average child age = 15.3 months old) • Mean age = 30.00 years • Predominantly low SES (*N* = 32)	• Non-English speaking • Insufficient cognitive capacity • Significant substance abuse	• Child social-emotional development • Anxiety • BPD • Maternal coping strategies • Depression • Reflective functioning • Parenting stress • Parenting sense of competence	• ASQ-SE2* • BAI* • BSL-23* • DBT-WCCL* • EPDS* • MSI-BPD* • NCAST* • PRFQ* • PSI-4SF* • PSOC*	Mother-Infant DBT. 25-weekly, 2.5-h DBT skills group program, adapted from evidence-based DBT. Conducted by mental health clinicians.	N/A	Significant improvements in anxiety, BPD symptoms, depression, and sense of parenting capabilities. No significant improvements in infant-parent interaction and the infant’s social-emotional development.
Wilson & Donachie (2018)	UK	RM	Referred internally	• *n* = 14 women (pregnant and postpartum) • Mean age = 31.00 years • All white British (100%)	• Not wanting to attend/unable to identify a goal/unable to commit to minimum of 8 sessions • Frequent high risk to life behaviours	• Psychological distress • Confidence in expressing/managing emotions • Mental health self-efficacy	• CORE-34* • LES* • MHCS*	Maternal emotional coping skills. 12-weekly, 2-h DBT skills group program. Facilitated by a consultant clinical psychologist and a perinatal specialist mental health nurse trained in DBT.	N/A	Significant decrease in psychological distress, and significant increases in confidence in expressing/managing emotions and mental health self-efficacy.
Yelland et al. (2015)	AUS	RM	Referred by clinician	• *n* = 89 mother-infant dyads (117 infants, average infant age = 7.82 months, 24 infants >12 months of age) • Mean age = 29.95 years	N/A	• Anxiety • Depression • Maternal postnatal attachment • BPD	• BAS* • Clinical interview (pre-test) • EPDS* • MPAS* • MSI-BPD*	Admission to an inpatient mother-baby unit, average length of stay = 22.34 days. Treatments include individual, group and mother-infant psychotherapy, medication, and electroconvulsive therapy. Supported by psychiatric, nursing and allied health staff.	N/A	Significant improvements in measures of anxiety, depression and BPD symptomatology. Mothers of infants younger than 12 months showed significant improvement in the mother-infant attachment relationship.

*Pre-test and post-test

**Pre-test, during, and post-test

***Pre-test, post-test, and follow-up

****Pre-test and follow-up

*****During and post-test

*Note*: *AAPI-2*, Adult-Adolescent Parenting Inventory-2; *AAI*, Adult Attachment Interview; *AMBIANCE*, Atypical Maternal Behaviour Instrument for Assessment and Classification; *ASQ-SE2*, Ages & Stages Questionnaire Social Emotional Index; *BAI*, Beck Anxiety Inventory; *BAS*, Beck Anxiety Scale; *BSI*, Brief Symptom Inventory Short Form; *BSL-2*3, Borderline Symptom List 23; *CES-D*, Center for Epidemiological Studies-Depression Scale; *CORE-34*, Clinical Outcomes in Routine Evaluation; *DBT-WCCL*, DBT-Ways of Coping Checklist; *DTS*, Davidson Trauma Scale; *EPDS*, Edinburgh Postnatal Depression Scale; *GAD-7*, Generalised Anxiety Disorder Questionnaire; *LES*, Living with Emotions Scale; *LSC*, Life Stressor Checklist; *MBQS*, Maternal Behaviour Q-sort; *MFA*, Maternal-Fetal Attachment; *MHCS*, Mental Health Confidence Scale; *MPAS*, Maternal Postnatal Attachment Scale; *MSI-BPD*, McLean Screening Instrument for BPD; *NCAST*, Nursing Child Assessment Satellite Training; Teaching Scale 2nd Ed; *PAI-BFS*, Personality Assessment Inventory - Borderline Features Scale; *PBI*, Parental Bonding Instrument; *PBQ*, Postnatal Bonding Questionnaire; *PRFQ*, Parental Reflective Functioning Questionnaire; *PSI-4SF*, Parenting Stress Index – short form; *RCT*, Randomised Controlled Trial; *RM*, Repeated Measures; *PSOC*, Parenting Sense of Competence Scale; *SSP*, Strange Situation Procedure; *TAS-20*, Toronto Alexithymia Scale

### Assessment of pathology and outcomes

All publications in the present review employed self-report measures to determine the presence of maternal pathology, with three studies concurrently using clinical interviews (Moran et al., 2005; Sadler et al., 2013; Yelland et al., 2015). BPD was measured by the McLean Screening Instrument for BPD (Zanarini et al., 2003) in two studies (Sved Williams et al., 2021; Yelland et al., 2015), and the Borderline Symptom List 23 scale (Bohus et al., 2009) in one study (Sved Williams et al., 2021). Emotional dysregulation was measured by the Toronto Alexithymia Scale (Bagby et al. 1994) in one study (Freethy et al., 2020), and the Living with Emotions Scale (Clarke & Wilson, 2008) in one study (Wilson & Donachie, 2018). Complex trauma was measured by the Life Stressor Checklist (Gray et al., 2004) in one study (Lavi et al., 2015), and the Adult Attachment Interview (George et al., 1985) in one study (Moran et al., 2005). To determine the presence of complex trauma symptomatology, one study (Sadler et al., 2013) utilised a pre-test pregnancy interview and considered demographic variables including age and socioeconomic status.

Like Moran et al. (2005), two additional studies included pre-test measures of maternal attachment, using the Maternal Fetal Attachment Scale (Cranley, 1981) (Lavi et al., 2015), and the Parental Bonding Instrument (Parker et al., 1979) (Sadler et al., 2013). Attachment as an outcome measure was used in four studies. Freethy et al. (2020) used the Postnatal Bonding Questionnaire (Brockington et al., 2001) at three timepoints (pre-test, post-test, and follow-up), and Yelland et al. (2015) used the Maternal Postnatal Attachment Questionnaire (Condon & Corkindale, 1998) pre- and post-test. Both RCTs (Moran et al., 2005; Sadler et al., 2013) utilised the Strange Situation procedure (Ainsworth et al., 1978) as an outcome measure of infant attachment.

### Evidence for dialectical behaviour therapy group skills

Recognised as an effective intervention for individuals with BPD, Dialectical Behaviour Therapy (DBT) encompasses the practice of mindfulness, distress tolerance, interpersonal effectiveness, and emotion regulation (Linehan, 2015). The therapy consists of four components: individual therapy, group skills training, therapist consultation team, and phone coaching. Only the group skills training component has been evaluated with perinatal BPD. Three of the repeated measures studies in the current review used perinatal modifications to the DBT group skills training manual (Freethy et al., 2020; Sved Williams et al., 2021; Wilson & Donachie, 2018).

Wilson and Donachie (2018) implemented Maternal Emotional Coping Skills over 12 weeks. In a mixed sample of 14 pregnant and postpartum women receiving care from a perinatal community mental health team, cPTSD was the most common diagnosis. Significant improvements with large effect sizes were found pre- to post-test for three outcome measures: psychological distress measured with the Clinical Outcomes in Routine Evaluation (Evans et al. 2000), *t* (13) = 5.32, *p* < .001; Cohen’s *d* = 0.83; mental health confidence and self-efficacy measured with the Mental Health Confidence Scale (Carpinello et al., 2000), *t* (13) = −8.03, *p* < .001, Cohen’s *d* = 0.83; and managing emotions measured with the Living with Emotions Scale (Clarke & Wilson, 2008), *t* (13) = −9.42, *p* < .001, Cohen’s *d* = 0.93.

Freethy et al. (2020) also implemented a DBT group skills program over 12 weeks in a mixed perinatal sample consisting of one pregnant and four postpartum women. Just two participants presented with emotional dysregulation, with the remaining three having mood disorders. Contrary to earlier findings (Wilson & Donachie, 2018), no significant differences in emotional regulation were found from pre- to post-test or follow-up, nor were significant differences found for postnatal bonding. Conclusions remain ambiguous given a lack of statistical power.

Sved Williams et al. (2021) examined Mother-Infant DBT, a 25-week program in 69 mothers with a clinical diagnosis of BPD or substantial BPD symptomatology. Recognising the extended definition of the perinatal period, a practical component was included for participants to exercise skills with their infant up to the age of three. Supporting previous findings on cPTSD (Wilson & Donachie 2018), significant pre- to post-test improvements in BPD symptomatology were found with moderate effect sizes (*r* = .35 to .36). A significant reduction in dysfunctional behaviours with moderate effect sizes was also found for maladaptive general dysfunction (*r* = 0.46) and blaming others (*r* = 0.42) measured with the DBT-Ways of Coping Checklist (Ne Neacsiu et al., 2010).

Significant improvements with moderate effect sizes were also found for parenting capability (*r* = 0.40) and reflective functioning (*r* = 0.29), the capacity to understand the infant’s mental state, using the Parenting Sense of Competence scale (Johnston & Mash, 1989), and interest and curiosity in mental states (*r* = 0.42) according to the Parental Reflective Functioning Questionnaire (PRF; Luyten et al., 2017). Just the “parent-child dysfunctional interaction” subscale on the Parenting Stress Index (Abidin, 2012) was significant post-test with a moderate effect size (*r* = 0.30). No significant differences were found for mother-infant interaction quality according to Nursing Child Assessment Satellite Training (NCAST; Oxford & Findlay, 2013) video recording ratings, or child social-emotional development.

### Evidence for home-visiting interventions

Two RCTs used home-visiting programs with adolescent perinatal samples presenting with symptoms of complex trauma (Moran et al., 2005; Sadler et al., 2013). Moran et al. (2005) trialled eight hourly home intervention sessions to promote maternal sensitivity with 50 mother-infant dyads when the infant was between 7 and 12 months old. The intervention was feedback provided to mothers upon viewing a 5-min video recording of their dyadic play interaction. The comparison group comprised 50 adolescent mother-infant dyads receiving one visit when the infant was 9 months old. According to the Strange Situation Procedure (Ainsworth et al., 1978), significantly more infants in the intervention group were securely attached at 12 months (*n* = 28, 57%), compared to the control group (*n* = 19, 38%), with a medium effect size (*W* = .25). No significant group differences were found for disorganised attachment style for infants in the intervention group (*n* = 28, 54%) and the control group (*n* = 29, 58%).

Significantly higher levels of sensitivity were seen in mothers in the intervention group (76%) in comparison to mothers in the control group (54%) at 24 months according to Maternal Behaviour Q-sort (Pederson et al., 1998) observations. However, no significant differences in sensitivity were found earlier between groups at 12 months. Further analyses revealed that the intervention did not benefit mothers classified as having an unresolved/disoriented mind state according to the Adult Attachment Interview (George et al., 1985). Thus, the home-visiting intervention was associated with increased likelihood of secure infant attachment, and maternal sensitivity at 24 months in adolescent mothers experiencing complex trauma. The main limitation of the design was the lack of matching of time for each condition; hence, we are unable to conclude if more attention alone may have produced superior results.

Sadler et al. (2013) examined Minding the Baby, an intensive home-visiting program based on mentalisation, nurse home visiting, nurse-family partnership, and infant-parent psychotherapy models. Weekly hourly visits were held from the third trimester until the infant was 1 year old, followed by biweekly visits until the age of two. The intervention group comprised 60 mother-infant dyads, and the control group comprised 45 mother-infant dyads. Control group members received routine prenatal and postnatal care including visits according to the community health centre clinical guidelines and received ongoing phone and mail contact for research scheduling purposes. Twelve-month immunisation schedules were significantly more likely to be adhered to in the intervention group in comparison to the control group; however, both groups were up to date by 24 months. Mothers in the intervention group had significantly lower rates of rapid subsequent childbearing (*n* = 1; 1.6%) compared to the control group (*n* = 7; 15%) (*p* = .019). No significant differences were found for child protective service referrals between groups.

At 4 months, there were no significant group differences in mother-infant affective communication. According to the Strange Situation Procedure (Ainsworth et al., 1978), more infants in the intervention group were securely attached at 12 months (*n* = 41, 64.4%), compared to the control group (*n* = 30, 48.4%). Supporting earlier findings (Moran et al., 2005), this difference was significant (odds ratio = 0.29, 95% CI = 0.10–0.88). Significant improvements in parental reflective functioning were only seen at 24 months for mothers in the intervention group with very low (<3) reflective functioning at their initial pregnancy interview. As with the Moran et al. (2005) study, the lack of clear matching of duration of each intervention makes it difficult to conclude that superior results were not simply the result of more attention.

### Evidence for child-parent psychotherapy

Lavi et al. (2015) examined a perinatal adaptation to Child-Parent Psychotherapy, an intervention designed to address consequences of trauma on wellbeing and safety by addressing maladaptive caregiving behaviours (Lieberman et al., 2011). Weekly sessions (*n* = 27) were carried out until the infant was 6 months old in a sample of 64 pregnant women with complex trauma and current intimate partner abuse. A significant pre- to post-test decrease was found in post-traumatic stress symptoms according to the Davidson Trauma Scale (Davidson et al., 1997) (Cohen’s *d* > .99). Significant increases in child-rearing attitudes were also seen according to the Adult-Adolescent Parenting Inventory-1 (Bavolek & Keene, 2001) (Cohen’s *d* = .95). Moderation analyses revealed that mothers initially presenting with low maternal-fetal attachment experienced the greatest improvements.

### Evidence for admission to an inpatient mother-baby unit

Yelland et al. (2015) reported on the clinical characteristics and outcomes for 89 mothers admitted to an inpatient mother-baby unit (MBU) accepting mothers with children up to the age of three. With an average length of stay of 22.34 days, interventions included individual, group, and mother-infant psychotherapy, medication, and electroconvulsive therapy. A significant improvement from admission to discharge was found for mothers presenting with BPD symptomatology (MSI-BPD; Zanarini et al., 2003). Significant improvements in the mother-infant attachment relationship were found in mothers with infants younger than 12 months according to the Maternal Postnatal Attachment Scale (Condon & Corkindale, 1998).

### Risk of bias

**Fig. 2:**
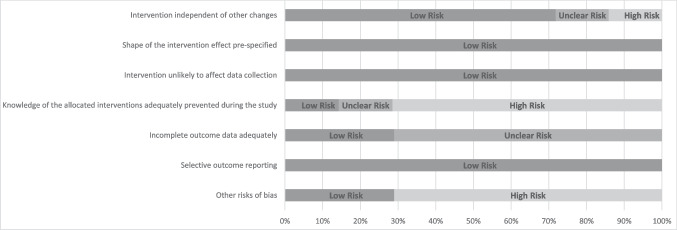
EPOC RoB by domain expressed as percentages across all studies included in the current review

Potential methodological issues were considered for each study according to the EPOC RoB tool. Both RCTs were included for comparative purposes, although the disparity in design quality is acknowledged. The authors had 88% agreement in ratings, with conflicts discussed until a consensus was reached. A low RoB for domains “Shape of the intervention effect pre-specified”, “Intervention unlikely to affect data collection”, and “Selective outcome reporting” was found. A low RoB was also found for “Intervention independent of other changes” excluding two studies, one with a high RoB (Freethy et al., 2020), and an unclear RoB (Wilson & Donachie, 2018). An unclear RoB was found for “Incomplete outcome data adequately”, excluding two studies with a low RoB (Sved Williams et al., 2021; Yelland et al., 2015). A high RoB was assigned to domain “Knowledge of the allocated interventions adequately prevented during the study” for the five repeated measures studies, and “Other risks of bias” excluding two studies with a low RoB (Sadler et al., 2013; Yelland et al., 2015). Figure 2 shows a summary of EPOC RoB by domain expressed as percentages.

## Discussion

The current systematic review synthesised the available literature on perinatal interventions for BPD, cPTSD, complex trauma, and emotional dysregulation. Publications described interventional studies including DBT group skills, home-visiting programs, Child-Parent Psychotherapy, and MBU admission. Although there exists a paucity of supporting literature, the quantitative analysis conducted thus far indicates the potential utility of these interventions in addressing outcomes pertaining to maternal mental health, BPD/trauma-related psychopathology, attachment, and parenting capability.

### Summary of effectiveness

In the absence of a comparison group, a DBT group skills intervention was associated with improvements in psychological distress, mental health confidence, self-efficacy, management of emotions, dysfunctional behaviours, and interest and curiosity in mental states. DBT group skills and MBU admission were associated with remission in BPD symptomatology, while Child-Parent Psychotherapy was associated with reduced post-traumatic stress symptomatology. Although there is considerable empirical support for DBT as an effective treatment for BPD, only two uncontrolled studies exist treating BPD perinatally and further research on the perinatal period is required using robust designs.

MBU admission was associated with improved maternal postnatal attachment, and home-visiting programs were associated with higher rates of securely attached infants (Moran et al., 2005; Sadler et al., 2013). Thus, results in the current review present preliminary evidence for MBUs and home-visiting programs for promoting healthy attachment relationships. However, neither of these studies uses time-matched attentional comparisons; hence, greater exposure to therapists may present as a confounding explanation for these promising findings.

Home-visiting programs were associated with less disrupted mother-infant interactions for teenaged mothers at 4 months, improved reflective functioning in high-risk mothers at 24 months, and higher sensitivity in adolescent mothers at 24 months. Improved reflective functioning was also seen with DBT group skills, as was sense of parenting competence according to self-report measures. As improvements in interaction quality were not found via objective ratings from DBT group skills, further evidence is required.

In summary, results in the current review suggest that further investigation of perinatal interventions is warranted, including DBT group skills, MBU admission, and Child-Parent Psychotherapy for perinatal mental health and remission of maternal BPD symptomatology; MBUs and home-visiting programs for promoting healthy attachment relationships; and home-visiting programs and DBT group skills for enhancing parenting capability. Further research on interventions is necessary to inform clinical guidelines due to the limited supporting literature.

### Study quality

The nascent field of treatment in new mothers with BPD/trauma contains few studies with robust research designs, with numerous limitations evident. The RoB summary in the current study describes varied results, with no study considered to have a low RoB for all domains. Insufficient statistical power due to small sample sizes, lack of objective measures, significant variation in wait times, and high attrition rates are noted. Regarding the latter, higher attrition rates are expected given the difficulties of engagement with this cohort (Dunn et al., 2020; National Institute for Health and Care Excellence [NICE], 2020). Only two RCTs were included in the current review, limiting any robust conclusions about whether the interventions were any better than the impact of time alone. Comparison across studies was also difficult, given that the Strange Situation Procedure (Ainsworth et al., 1978) was the only overlapping measure in the RCTs, and Moran et al. (2005) did not explicitly state whether this was assessed blindly, thereby potentially biasing outcomes. To increase ecological validity in future research, it is suggested that an additional measure of infant attachment be utilised, such as the Attachment Q-Sort (Waters & Deane, 1985).

Heterogeneity of test measures for assessing psychopathology is noted in the current review, as is heterogeneity regarding stage of perinatal period within and between studies for participants. In non-clinical samples, there are high prevalence rates of BPD symptomatology in pregnant women (6.9–26.7%; Prasad et al., 2022), yet postpartum prevalence rates remain unknown. As traumatic memories can be triggered by the anticipation of childbirth (Blankley et al., 2015; De Genna et al., 2012; Newman, 2015), symptom remission could be seen upon completion of labour. Conversely, symptomatology could present in women with personality vulnerability when engaging in postpartum caregiving requirements, such as soothing a crying infant (Geerling et al., 2019). Hence, varied sample characteristics due to perinatal stage could have compromised conclusions drawn from comparative analysis in the current review.

Further to limitations due to sample characteristics, the exclusion criteria of two studies (Lavi et al., 2015; Wilson & Donachie, 2018) may have failed to adequately represent the target population. This included current use of alcohol and/or drugs, and frequent high risk to life behaviours such as “cutting, overdosing or endangering life including excess use of alcohol and/or non-prescribed medication”. Given BPD/trauma is frequently characterised by substance use and self-harm (Dunn et al., 2020; Nagel et al., 2021; Sved Williams & Apter, 2017), sample representativeness and consequently outcomes may be questionable. It is additionally noted that some BPD/trauma behaviours are incompatible with eastern and collectivist societies, for example substance use and promiscuous sex in China (Wang et al., 2012). As BPD and cPTSD are not cross-culturally recognised disorders, perinatal screening and intervention may be inappropriate. Hence, as all studies were conducted in western societies, cultural homogeneity of sample characteristics is noted.

Finally, although “BPD/trauma” was used in the present review to encompass BPD and cPTSD/complex trauma, consideration of the clinical distinctions between constructs is required. Generalisability of interventions may be limited, with just DBT group skills applied to both BPD (Sved Williams et al., 2021) and cPTSD (Wilson & Donachie, 2018). For example, interventions addressing fear of abandonment may be beneficial for mothers with BPD, whereas responding to symptoms of hyperarousal may be more appropriate for mothers with cPTSD. Such limitations further underscore the necessity for further research in the field.

### Future research

The NICE (2020) guidelines suggest that structured clinical management (a psychologically informed model of case management) may be a preferred intervention option in this field. Such may afford increased capacity and flexibility for women with BPD/trauma during the perinatal period. Emphasising a collaborative approach with the support of a generalist mental health care professional, skills include emotion management and problem solving. A RCT is recommended that compares two active conditions of case management and standard care measuring maternal and infant mental, physical, and interpersonal outcomes. This approach would appear to be more feasible in terms of resources and ability to be embedded in real-world settings. This comparison would benefit from inclusion of a health economic perspective to allow conclusions to be drawn about the costs versus the benefits of different approaches.

## Conclusion

The results of this systematic review present preliminary evidence that supports ongoing evaluation of the potential utility of four pilot perinatal interventions in addressing maternal mental health, BPD/trauma-related psychopathology, attachment, and parenting capability. Conclusions regarding overall efficacy of interventions is considerably restricted by limited quantity and quality of evidence, and ability to compare results across studies due to non-overlap of validated measures. Designed for pathological cases of higher severity, the interventions are quite intensive, involving multiple in-person facilitation. Hence, largescale feasibility of such interventions is dubious. Alternatively, future research could consider utilising antenatal screening to identify at-risk mothers, and subsequent implementation of early antenatal intervention such as case management. Given the rapid risks of maternal BPD/trauma on infant development, early intervention may be key to breaking the intergenerational cycle.

## Data Availability

As this is a systematic review, no research data beyond what is already currently available in databases PsycInfo, MEDLINE, Emcare, Scopus, and ProQuest Dissertations and Theses Global database was reported.
